# Novel Highly Pathogenic Avian Influenza A(H5N1) Virus, Argentina, 2025

**DOI:** 10.3201/eid3112.250783

**Published:** 2025-12

**Authors:** Ralph E.T. Vanstreels, Martha I. Nelson, María C. Artuso, Vanina D. Marchione, Luana E. Piccini, Estefania Benedetti, Alvin Crespo-Bellido, Agostina Pierdomenico, Thorsten Wolff, Marcela M. Uhart, Agustina Rimondi

**Affiliations:** University of California Davis School of Veterinary Medicine, Davis, California, USA (R.E.T. Vanstreels, M.M. Uhart); National Institutes of Health, Bethesda, Maryland, USA (M.I. Nelson, A. Crespo-Bellido); Servicio Nacional de Sanidad y Calidad Agroalimentaria, Martínez, Buenos Aires, Argentina (M.C. Artuso, V.D. Marchione, L.E. Piccini); Instituto Nacional de Enfermedades Infecciosas Dr. Carlos G. Malbrán, Buenos Aires (E. Benedetti); Servicio Nacional de Sanidad y Calidad Agroalimentaria, Buenos Aires (A. Pierdomenico); Robert Koch-Institut, Berlin, Germany (T. Wolff, A. Rimondi)

**Keywords:** Avian influenza, influenza, highly pathogenic avian influenza, H5N1, genotype B3.2, South American low pathogenicity avian influenza, viruses, reassortment, backyard poultry, viral genome, genetic shift, notifiable disease, respiratory infections, Argentina

## Abstract

Genomic sequencing of reemerging highly pathogenic avian influenza A(H5N1) virus detected in Argentina in February 2025 revealed novel triple-reassortant viruses containing gene segments from Eurasian H5N1 and low pathogenicity viruses from South and North American lineages. Our findings highlight continued evolution and diversification of clade 2.3.4.4b H5N1 in the Americas.

Highly pathogenic avian influenza (HPAI) viruses were introduced to South America in 2022 by migratory birds from North America. The viruses belonged to the 2.3.4.4b clade of HPAI A(H5N1) virus that became widespread in Europe in 2020 and spread to North America in 2021. The trajectory of H5N1 in South America has differed from H5N1 in North America in several critical ways. First, nearly all South America outbreaks stem from a single introduction of H5N1 viruses from North America (*1*,*2*), whereas the North America epizootic was reseeded by multiple independent introductions from Europe and Asia (A1–A6) (*3*,*4*). Second, South America H5N1 outbreaks were driven by a single genotype (B3.2) that was introduced from North America and remained genetically stable during its spread across South America. In contrast, H5N1 viruses in North America underwent frequent reassortment with low pathogenicity avian influenza (LPAI) viruses, prompting new genotype nomenclature (using B, C, D) (*3*). Third, South America’s H5N1 epizootic is unique in establishing mammal-to-mammal transmission in marine mammals, enabled by the H5N1 (B3.2) virus acquiring mammalian-adaptive polymerase basic (PB) 2 mutations (Q591K and D701N) (*1*,*2*). That pattern has not occurred in North America, where H5N1 spillover into terrestrial and marine mammals was transient, except in United States dairy cattle (*3*).

Beyond the ecologic devastation among coastal wildlife, in 2023, H5N1 (B3.2) virus spread widely in birds across mainland South America, leading to poultry and wild bird outbreaks (*5*–*8*). Although in 2024 HPAI outbreaks occurred in Brazil and Peru (World Organisation for Animal Health, https://wahis.woah.org), there were no detections in Argentina during March 2024–January 2025.

## The Study

On February 11, 2025, Servicio Nacional de Sanidad y Calidad Agroalimentaria (SENASA; Buenos Aires, Argentina), Argentina’s national organization for agricultural health and safety, was notified of an outbreak in a mixed backyard flock (chickens, ducks, and turkeys) in Chaco Province, northern Argentina. The flock experienced high mortality (33/81 chickens, 37/99 ducks) in just 1 week. When we inspected the living flock, two thirds of the remaining 48 chickens had diarrhea and 1 of the remaining 62 ducks was lethargic; 2 turkeys were asymptomatic. The household was located within a remnant fragment of the Dry Chaco biome, a hot and semi-arid tropical dry forest, surrounded by agriculture cropland. The affected flock had free access to a small pond frequently visited by wild waterfowl (Appendix 1). We depopulated and disinfected the area. We inspected backyard poultry within the 3 km perifocal zone (1 household) and the 3–10 km surveillance zone (7 households) and detected no illness or death. We did not find any affected wildlife on site.

We collected oropharyngeal and cloacal swab samples from 8 birds to test for influenza A virus. We tested and subtyped the samples by real-time PCR at SENASA. We performed next-generation sequencing on positive samples as previously described (*6*) (Appendix 1). We deposited full and partial genome sequences in the GISAID database (https://gisaid.org; accession nos. EPI_ISL_19752381 and EPI_ISL_19823059–68).

**Figure 1:**
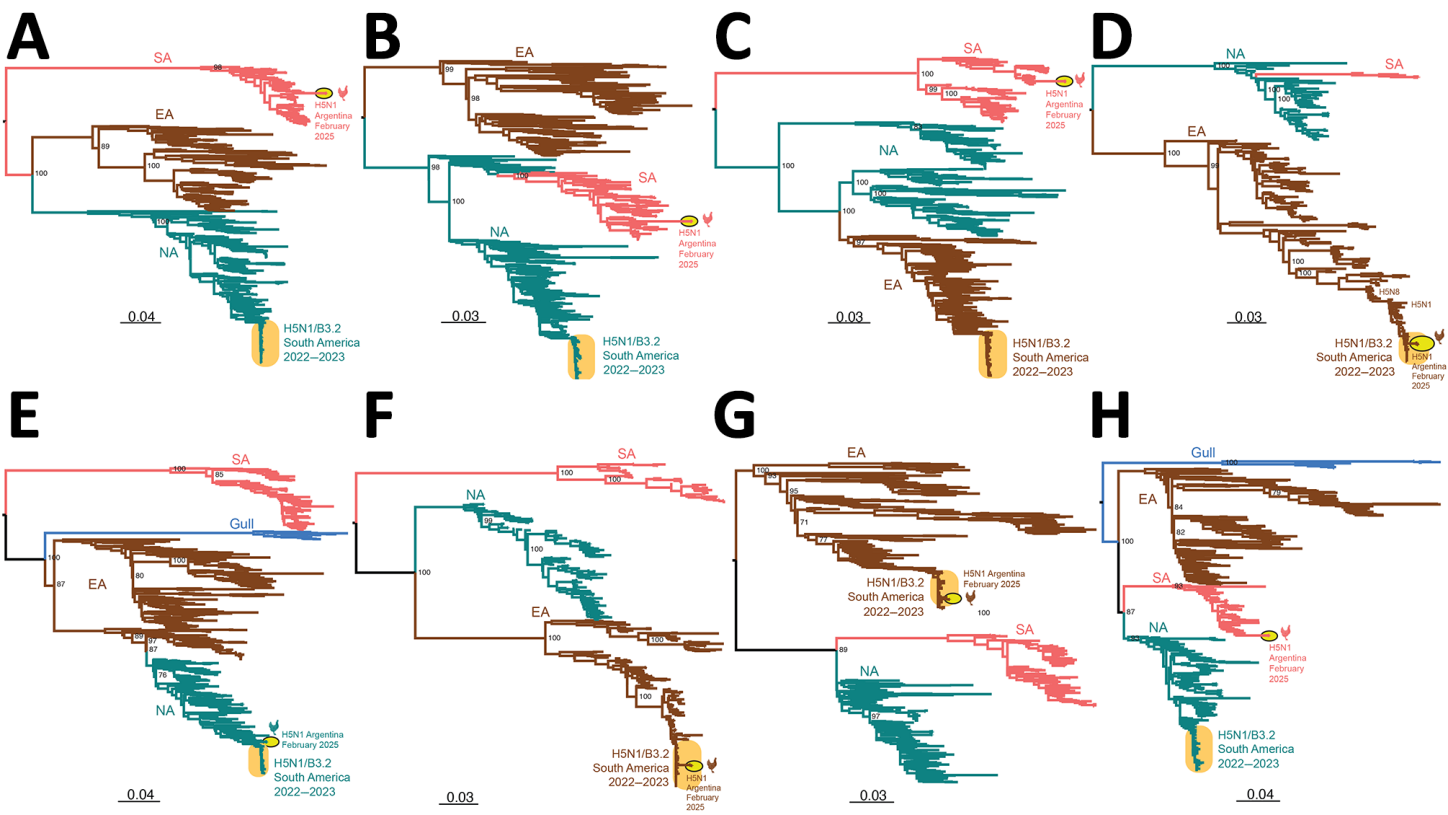
Maximum-likelihood trees inferred for the 8 genome segments sequenced in this study of novel triple reassortant highly pathogenic avian influenza A(H5N1) virus, Argentina, 2025. Phylogenetic trees were inferred by using IAVs collected globally in avian hosts. A) Gene segment PB2. B) Gene segment PB1. C) Gene segment PA. D) Gene segment H5. E) Gene segment NP. F) Gene segment N1. G) Gene segment MP. H) Gene segment NS; only A allele is shown. The number of sequences used to construct each tree was 676 for PB2, 667 for PB1, 686 for PA, 271 for H5, 682 for NP, 443 for N1, 639 for MP, and 506 for NS (Appendix 2, https://wwwnc.cdc.gov/EID/article/31/12/25-0783-App2.xlsx). Trees are midpoint rooted for clarity. Key node bootstrap values are shown. Teal shading represents NA lineage, brown shading represents EA lineage, pink shading represents SA lineage, and blue shading represents gull lineage. Orange oval represents previously reported SA H5N1 clade B3.2 genotype virus. Yellow oval with black outline and chicken silhouette represents the novel H5N1 virus found in Argentina. Branch lengths are drawn to scale. Scale bars represent nucleotide substitutions per site. EA, Eurasian; MP, matrix protein; NA, North American; NP, nucleoprotein; NS, nonstructural protein; PA, polymerase; PB, polymerase basic; SA, South American.

**Figure 2:**
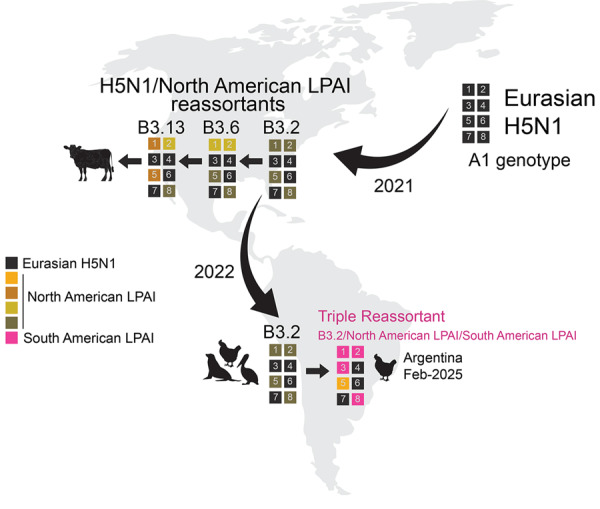
Key reassortment and migration events leading to the novel triple reassortant influenza A(H5N1) viruses in February 2025, Argentina. Each box represents 1 of the 8 segments of the influenza A virus genome, numbered in order of longest to shortest length: 1, polymerase basic protein 2; 2, polymerase basic protein 1; 3, polymerase acidic protein; 4, hemaggultinin; 5, nucleoprotein; 6, neuraminidase; 7, matrix protein; and 8, nonstructural protein. Curved black arrows indicate the direction of major geographic migration events. Straight black arrows indicate sequential reassortment events of interest. LPAI, low pathogenicity avian influenza.

**Figure 3:**
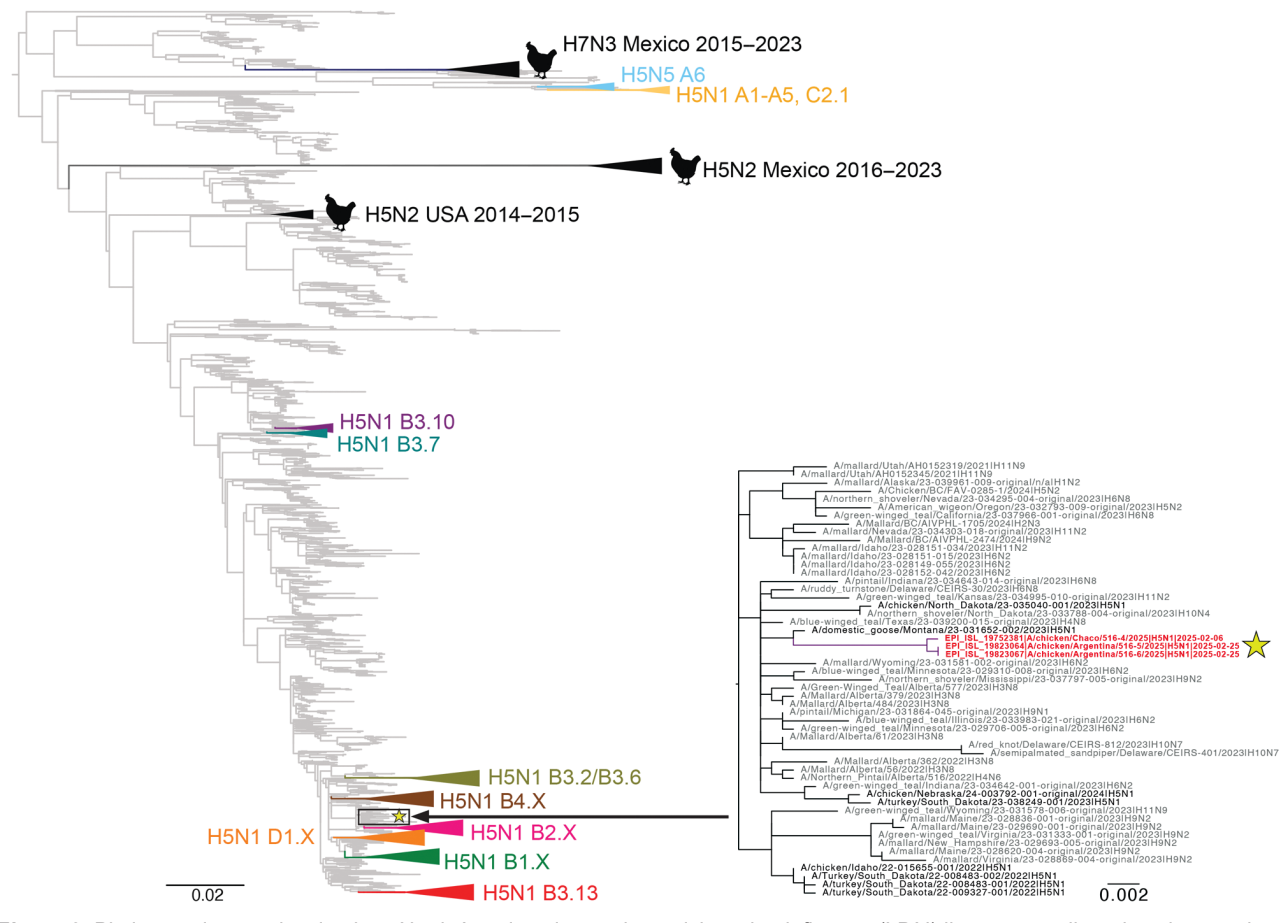
Phylogenetic tree showing how North American low pathogenicity avian influenza (LPAI) lineage contributed nucleoprotein (NP) genes by reassortment to novel influenza A(H5N1) viruses from Argentina, 2025. We inferred the phylogenetic tree by using the maximum-likelihood method for 11,820 North American LPAI and highly pathogenic avian influenza (HPAI) NP sequences collected during 2015–2025. Gray indicates LPAI viruses. HPAI H5N1 clades are collapsed and shaded in different colors and labeled according to corresponding H5 clade 2.3.4.4b genotypes. Black indicates prior H5N2 and H7N3 outbreaks in poultry. The 3 H5N1 viruses collected from poultry in Argentina in February 2025 are indicated in red and with a yellow star. A more detailed subsection of the tree containing those 3 viruses is shown, with tip labels. Branch lengths are drawn to scale. Scale bar represents nucleotide substitutions per site.

We inferred global avian influenza virus (AIV) phylogenies (including both HPAI and LPAI virus sequences) independently for each of the 8 gene segments to determine the genetic composition of H5N1 viruses from this outbreak (H5N1-Arg_Feb2025 viruses). The phylogenies indicated the H5N1-Arg_Feb2025 viruses are novel 4:3:1 triple reassortants (Figure 1; Appendix 1). Four gene segments (PB2, PB1, polymerase [PA], and nonstructural [NS]) belong to the South American LPAI lineage (Figure 1) that has circulated regionally in wild birds for decades (*9*–*12*). Three gene segments (hemagglutinin [HA], neuraminidase [NA], and matrix protein [MP]) clustered with H5N1 viruses of the B3.2 genotype and belong to the original Eurasian H5N1 lineage introduced to North America (Figure 2). We identified novel amino acid changes in the HA and NA segments of H5N1-Arg_Feb2025 viruses (Table) (*13*); the functional relevance of those changes is unknown but might merit further investigation if consistently detected in future outbreaks. One segment, nucleoprotein (NP), did not cluster with any previously known South American viruses and instead grouped with North American LPAI viruses (Figure 3). Because of limited surveillance in South America, where ≈90% of full-genome sequences are from Argentina and Chile, it was difficult to determine how the North America–derived NP segment became part of the triple reassortant H5N1 viruses we identified.

**Table:** Amino acid differences in hemagglutinin and neuraminidase of novel triple reassortant influenza A(H5N1) virus from Argentina, 2025, compared with previously reported South American highly pathogenic avian influenza A(H5N1) viruses

Protein (subunit)	Position*	Amino acid in previous viruses	Mutated amino acid in reassortant virus	Protein domain
Hemagglutinin (HA1)	64	E	D	Vestigial esterase domain
	172	L	Y	Receptor-binding domain
	181	S	P	Receptor-binding domain
	208	T	K	Receptor-binding domain
	235	P	S	Receptor-binding domain
Hemagglutinin (HA2)	493	E	K	Helical globule
	510	V	I	Transmembrane domain
	532	M	I	Cytoplasmic tail
Neuraminidase	35	S	V	Stalk domain
	49	C	R	Stalk domain
	68	N	Y	Head domain
	454	G	S	Stalk domain

*HA positions refer to H5 numbering.

## Conclusions

We have documented a reassortment event between HPAI H5N1 and endemic South America LPAI viruses. South American PB2 and PA segments are divergent from global AIV diversity (*9*) (Figure 1), indicating reassortment has expanded H5N1 polymerase diversity. Although the H5N1-Arg_Feb2025 viruses have exchanged 5 gene segments, they retained the original Eurasian MP segment (Figure 2), which remains conserved in most reassortant H5N1 viruses in North America. That segment conservation suggests the Eurasian MP segment might confer a selective advantage in HPAI H5 viruses. To date, we found no evidence of those novel 4:3:1 triple reassortant viruses in other South America countries; however, if future detections confirm wider spread, designation of a new H5N1 genotype would be warranted. Of consequence, genotyping tools such as the US Department of Agriculture’s GenoFlu should be expanded to include South American lineage genes for systematic classification of new virus genotypes.

Reassortment is a key mechanism in the evolution and host adaptation of AIV, often enabling their emergence in new animal communities and contributing to the development of strains with panzootic or pandemic potential (*14*,*15*). The genomic constellation of the H5N1-Arg_Feb2025 viruses parallels patterns found in North America, where clade 2.3.4.4b H5N1 viruses have also incorporated primarily internal LPAI genes. H5N1-Arg_Feb2025 viruses replaced the PB2, PB1, NP, and NS segments from the parental B3.2 genotype, a reassortment pattern observed in North American genotypes B3.6 and B3.13 (Figure 2), both derived from B3.2. However, the H5N1-Arg_Feb2025 viruses also replaced the Eurasian PA segment with a segment from South American LPAI viruses. Despite substantial genomic changes, the novel reassortant viruses from Argentina caused illness and death rates comparable to those previously observed for the B3.2 genotype. The primary clinical manifestation in this outbreak was diarrhea, affecting nearly two thirds of chickens. During H5N1 poultry outbreaks in Argentina in 2023, diarrhea was less common (reported in 29.7% of outbreaks), whereas high death rates (81.2%), lethargy (65.4%), cyanotic comb (57.4%), and neurologic signs (30.7%) were more frequent (n = 101 outbreaks) (SENASA, unpub. data). The predominance of gastrointestinal signs suggests possible shifts in tissue tropism or virulence. Also, the detection of a North American NP segment not previously identified in LPAI viruses from Argentina or elsewhere in South America highlights the need to strengthen regional AIV surveillance, even in the absence of active HPAI circulation. There is a lack of information on avian species and flyways involved in introductions of North American gene segments to South American LPAI viruses. Moreover, investigating the functional role of the North America–derived NP gene within the South American internal genes could clarify its potential contribution to the distinct gastrointestinal phenotype observed in this outbreak.

Further research on the diversity of LPAI viruses circulating in Neotropical wildlife will be essential to understand potential interactions between H5N1 and South American lineage strains and to assess the long-term consequences of the introduction of HPAI viruses into the region. Our findings underscore the critical importance of sustained influenza surveillance coupled with whole-genome sequencing to track the evolution of HPAI H5N1 and support efforts to control and mitigate its effect on domestic animals, wildlife, and human health. 

Appendix 1 Additional information about novel triple reassortant highly pathogenic avian influenza A(H5N1) virus, Argentina, 2025.

Appendix 2 Sequence lists used to create phylogenetic trees in this study of novel triple reassortant highly pathogenic avian influenza A(H5N1) virus, Argentina, 2025.
