# Supplementary material for: Novel Highly Pathogenic Avian Influenza A(H5N1) Virus, Argentina, 2025
Source: Emerg Infect Dis. 2025 Dec;31(12):2279–83. doi: 10.3201/eid3112.250783 (PMC12782262; doi:10.3201/eid3112.250783)
Supplement: Appendix 1 — Additional information about novel triple reassortant highly pathogenic avian influenza A(H5N1) virus, Argentina, 2025. [file 25-0783-Techapp-s1.pdf]

Article DOI: https://doi.org/10.3201/eid3112.250783

EID cannot ensure accessibility for supplementary materials supplied by authors. Readers who have difficulty accessing supplementary content should contact the authors for assistance.

Novel Highly Pathogenic Avian Influenza A(H5N1) Virus, Argentina, 2025

Appendix 1

Details on the laboratory methods employed for the detection and full-genome characterization of HPAI H5N1 viruses in samples from backyard poultry in Chaco province, northern Argentina, February 2025.

Virus detection

Viral RNA was extracted from individual swabs of one duck and seven chickens for IAV detection and subtype determination (Appendix Table). Briefly, RNA was extracted from 500 µL of suspension with RNeasy® Mini Kit (Qiagen, Hilden, Germany). RNA was eluted in a final volume of 50 µL and AIV was tested by M-gene real-time RT-PCR and subtyped by real-time RT-PCR (Real Time PCR 7500; Applied Biosystems, Waltham, USA) following the protocols from SENASA according to the standard operating protocols from NVSL-USDA.

Full genome sequencing

The viral genome was amplified from RNA using a multi-segment one-step RT-PCR with Superscript III high-fidelity RT-PCR kit (Invitrogen, Carlsbad, USA) according to manufacturer's instructions using the Opti1 primer set (Opti1-F1, Opti1-F2 and Opti1-R1) as previously described (*1*). The RT-PCR amplification parameters were: 2 min at 55°C, 60 min at 42°C, and 2 min at 94°C, followed by 5 cycles of (94°C/30 s; 44°C/30 s; 68°C/3.5 min), 26 cycles of (94°C/30 s; 57°C/30 s; 68°C/3.5 min), and a final extension for 10 min at 68°C. Amplicons were visualized on a 1% agarose gel and purified with Agencourt AMPure XP beads (Beckman Coulter, Brea, USA). The concentration of purified amplicons was quantified using the Qubit High Sensitivity dsDNA kit and a Qubit Fluorometer (Invitrogen). Samples were

sequenced in a NovaSeq 6000 platform (Illumina, San Diego, USA). Contig assembly was conducted using the Iterative Refinement Meta-Assembler v.1.0.3 (*2*).

Phylogenetic analysis

Publicly available sequences (as of May 2025) were obtained from GenBank (https://www.ncbi.nlm.nih.gov/genbank) and the Global Initiative on Sharing All Influenza Data (https://gisaid.org). Globally available sequences from influenza A viruses from avian hosts were included. Sequences were aligned with MAFFT v7.520 (https://mafft.cbrc.jp/alignment/software) and Maximum likelihood (ML) phylogenetic trees were inferred for each of the eight genome segments using the ML methods available in IQ-Tree 2 (*3*), with a GTR model of nucleotide substitution with gamma distributed rate variation among sites. Due to the size of the dataset, we used the high-performance computational capabilities of the Biowulf Linux cluster at the National Institutes of Health (http://biowulf.nih.gov). To assess the robustness of each node, a bootstrap resampling process was performed with 1000 replicates.

Appendix Table. Cq values obtained by RRT-PCR from samples from backyard poultry in Chaco province, northern Argentina, 2025.

Host	Swab	Cq (IAV)	Cq (H5) AM/EU	Cq (H5) Gs/Gd	Cq (Pathotype)	Isolate Name
Duck	Oropharyngeal	34.46	27.49	ND*	26.07	A/Duck/Argentina/516–3/2025
Chicken	Oropharyngeal	29.39	21.65	24.18	21.88	A/Chicken/Argentina/516–4/2025
Chicken	Oropharyngeal	26.17	20.56	21.78	19.07	A/Chicken/Argentina/516–5/2025
Chicken	Oropharyngeal	26.11	21.09	21.61	18.78	A/Chicken/Argentina/516–6/2025
Chicken	Oropharyngeal	25.67	19.71	20.26	17.44	A/Chicken/Argentina/516–7/2025
Chicken	Oropharyngeal	23.91	17.89	21.22	15.36	A/Chicken/Argentina/516–8/2025
Chicken	Cloacal	35.24	29.79	30.12	27.16	A/Chicken/Argentina/516–9/2025
Chicken	Cloacal	22.78	27.26	26.82	24.35	A/Chicken/Argentina/516–10/2025

*ND = Not determined

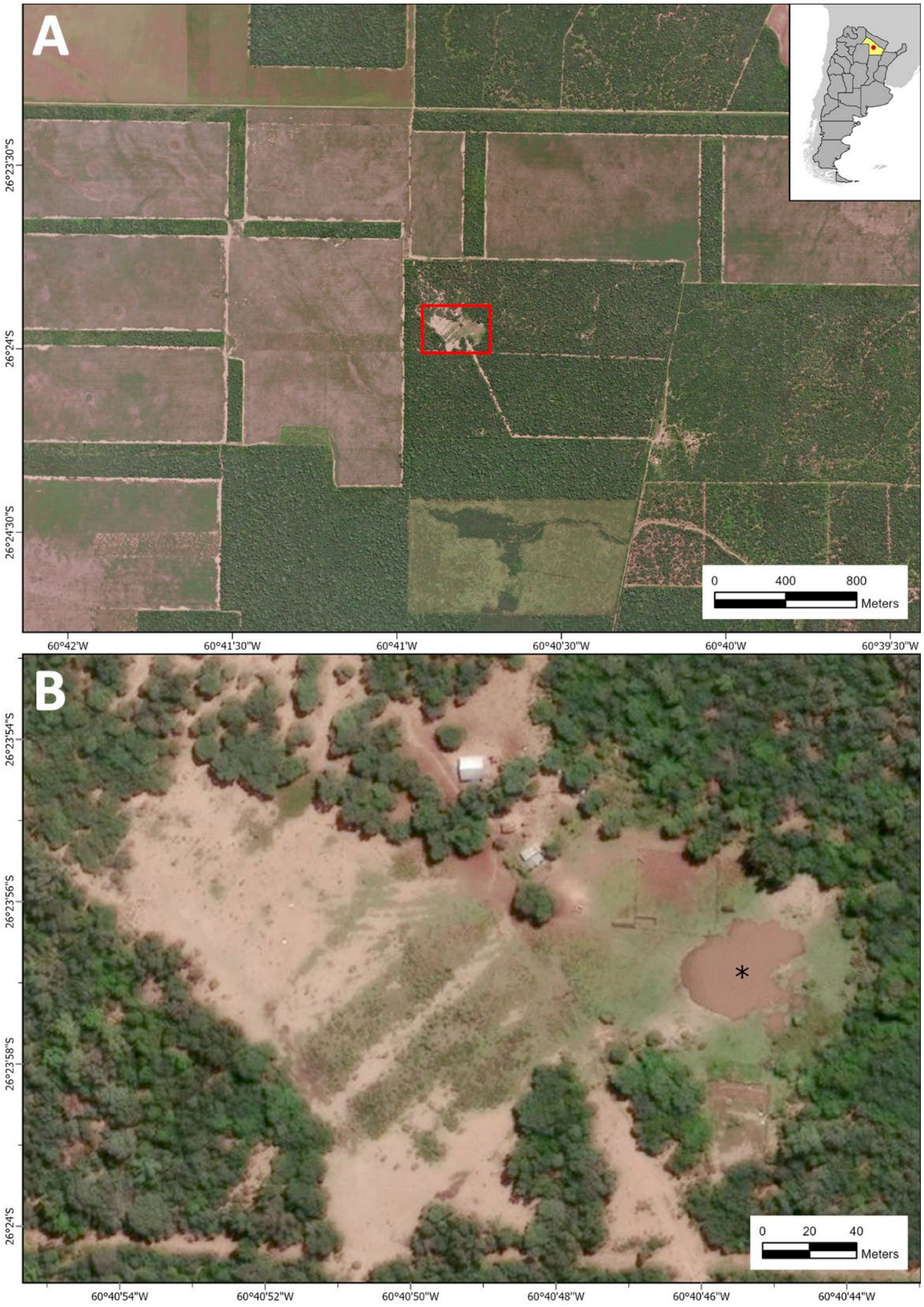

Appendix Figure 1. Overview of the mosaic landscape of Dry Chaco forest and agriculture cropland where an HPAI H5N1 outbreak was recorded in backyard poultry in Argentina (26.3993°S, 60.6797°W), February 2025. Chaco province is drawn in yellow in the map of Argentina (top right). The affected flock had access to a small pond (40 × 30 m) frequently visited by wild waterfowl (asterisk).

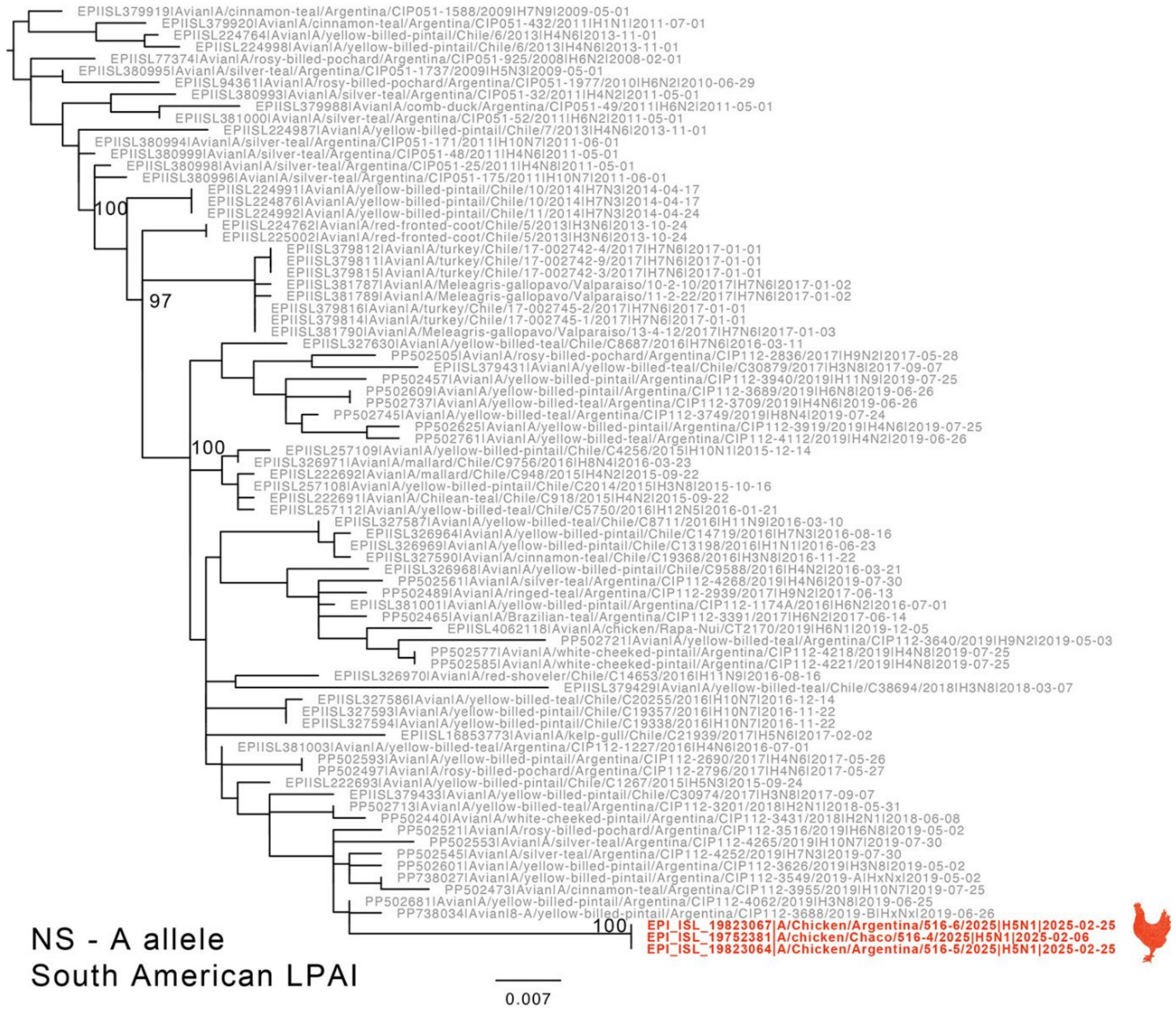

Appendix Figure 2. Zoomed view of the PB2 segment of the South American low pathogenic avian influenza virus phylogenetic tree showing the position of H5N1-Arg_Feb2025 virus.

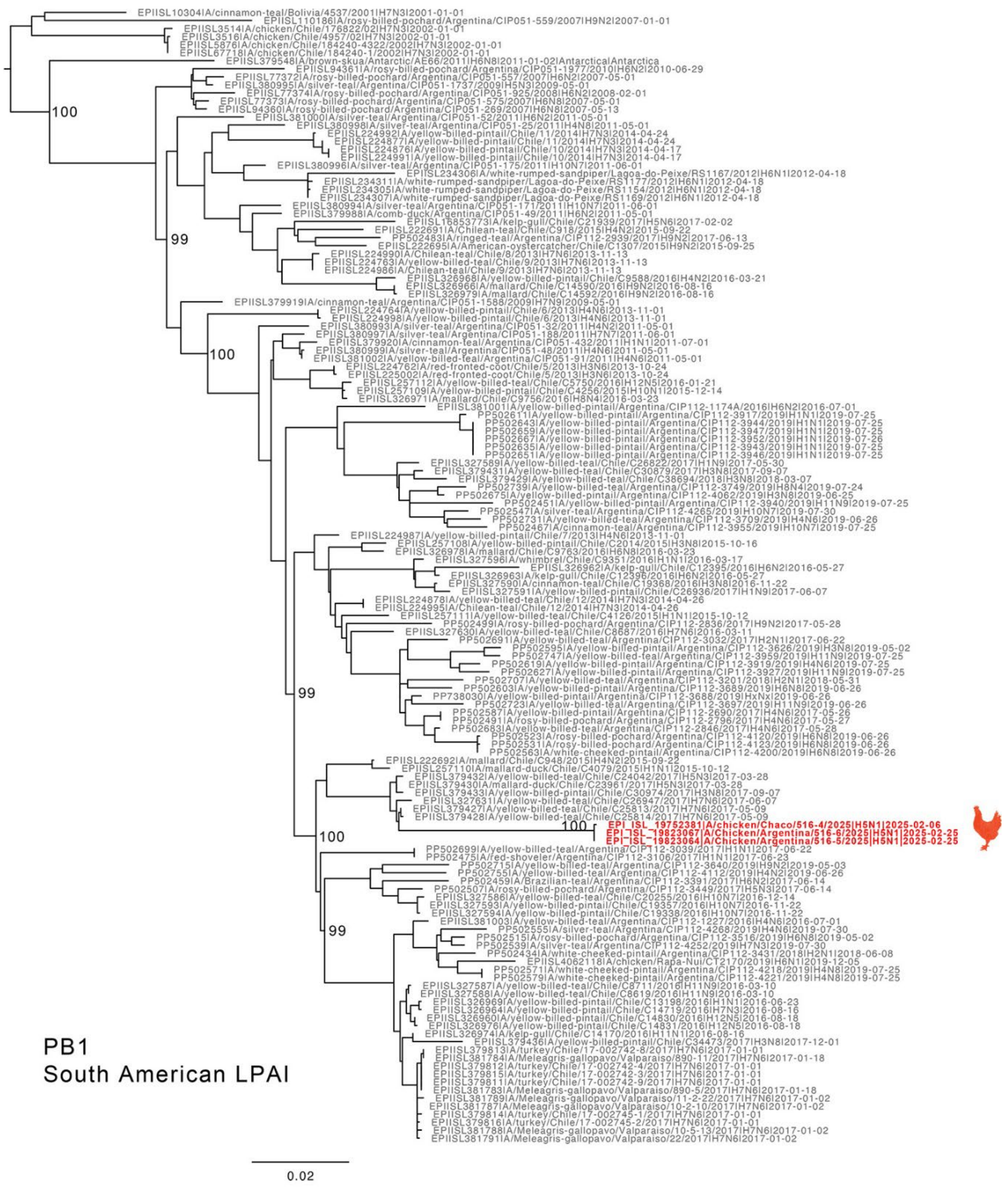

Appendix Figure 3. Zoomed view of the PB1 segment of the South American low pathogenic avian influenza virus phylogenetic tree showing the position of H5N1-Arg_Feb2025 virus.

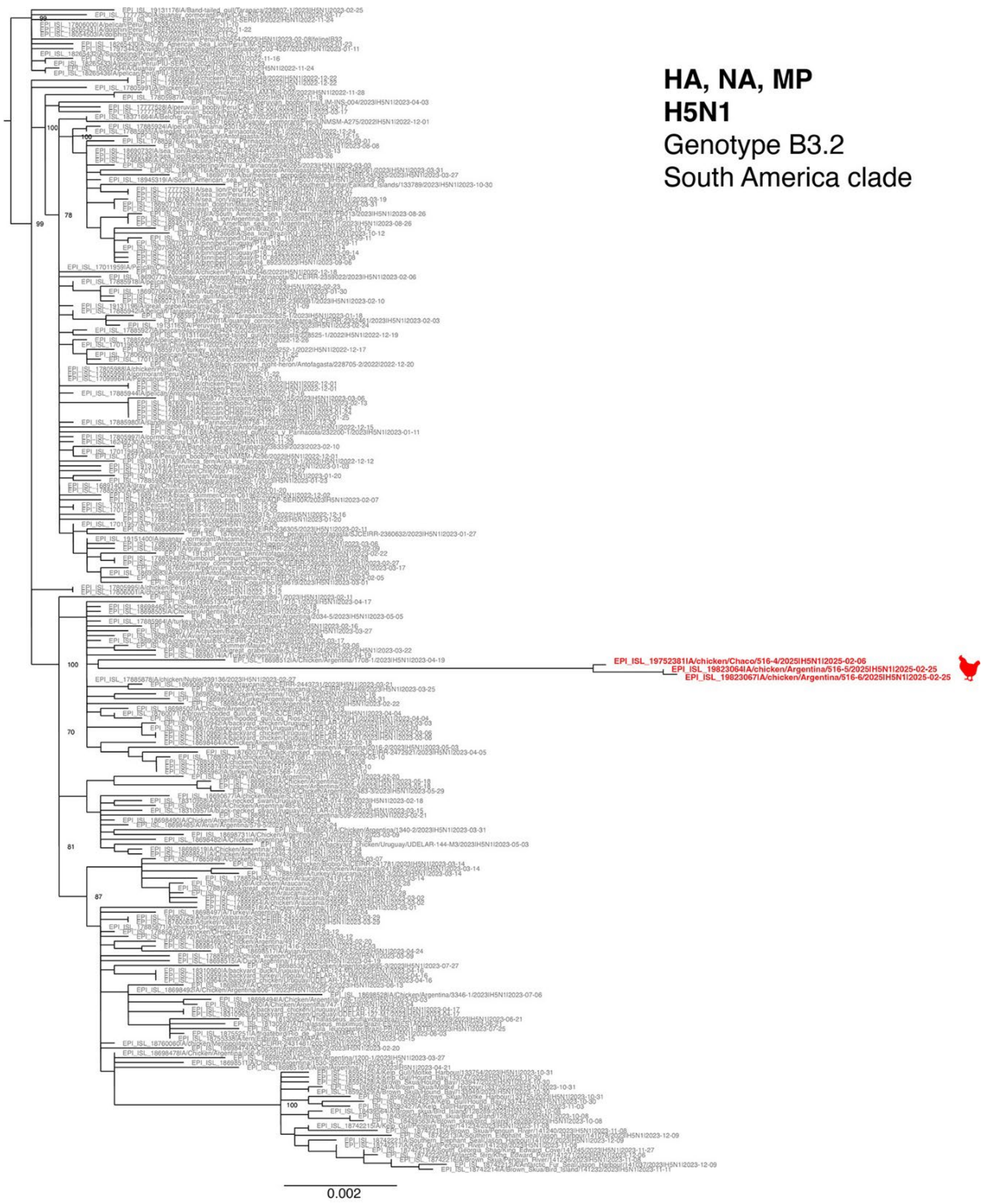

Appendix Figure 4. Zoomed view of the PA segment of the South American low pathogenic avian influenza virus phylogenetic tree showing the position of H5N1-Arg_Feb2025 virus.

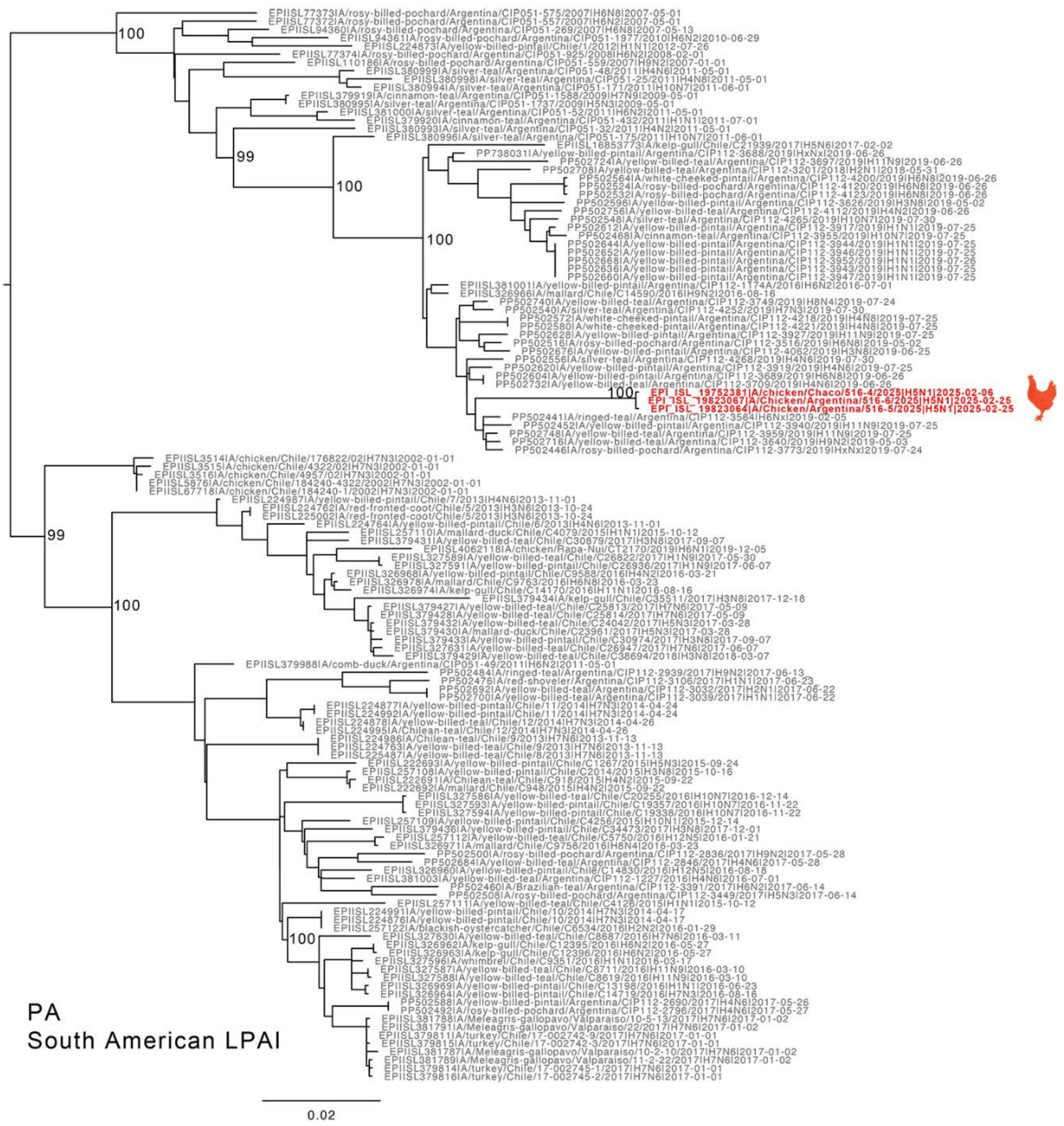

Appendix Figure 5. Zoomed view of the HA, NA, MP segments of the H5N1 Genotype B3.2 South American clade influenza virus phylogenetic tree showing the position of H5N1-Arg_Feb2025 virus.

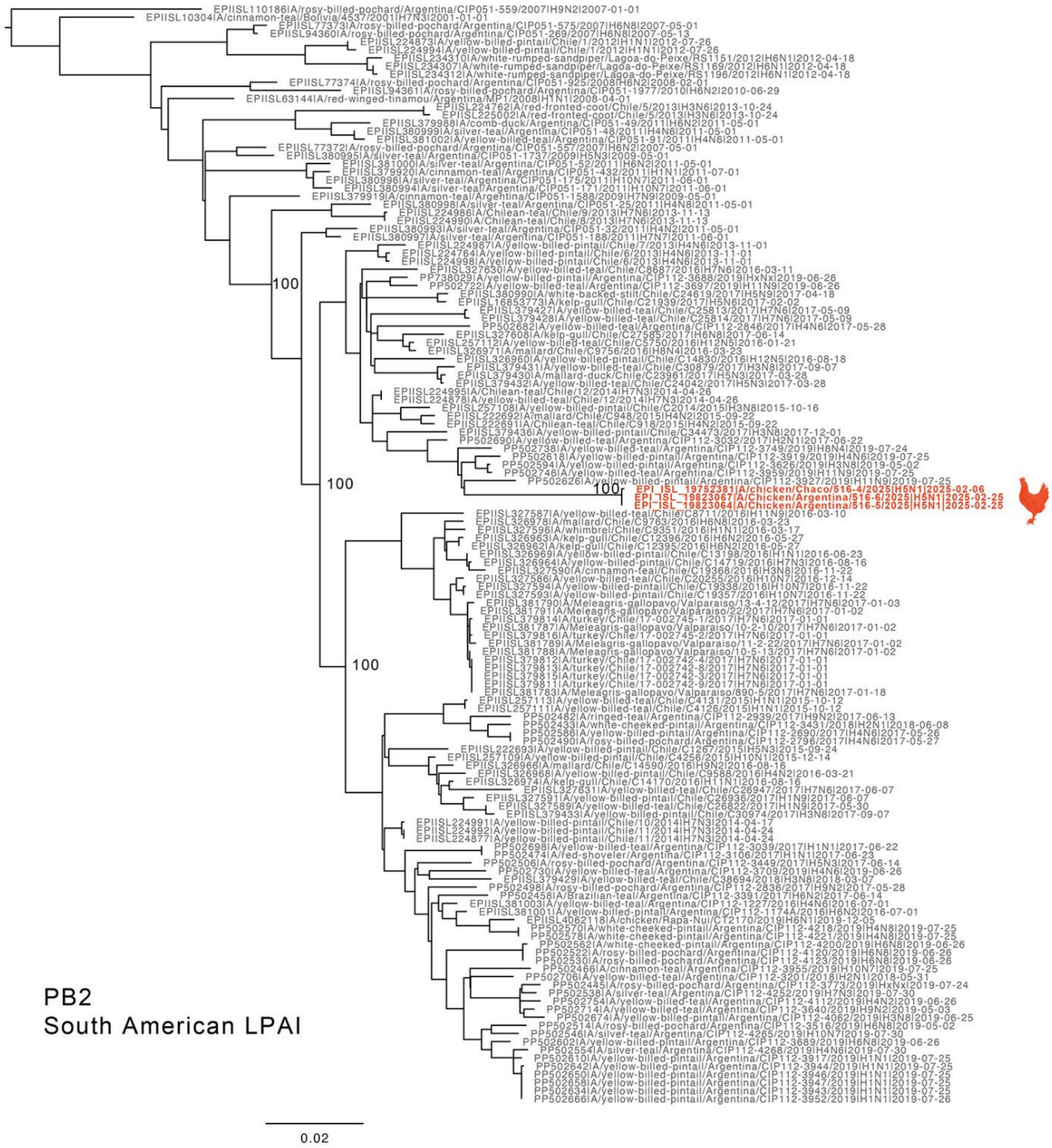

Appendix Figure 6. Zoomed view of the NS A allele segment of the South American low pathogenic avian influenza virus phylogenetic tree showing the position of H5N1-Arg_Feb2025 virus.

References

1. Zhou B, Wentworth DE. Influenza A Virus Molecular Virology Techniques. In: Kawaoka Y, Neumann G, editors. Influenza virus: methods and protocols. Vol. 865. Totowa, NJ: Humana Press; 2012. p. 175–92.

2. Shepard SS, Meno S, Bahl J, Wilson MM, Barnes J, Neuhaus E. Viral deep sequencing needs an adaptive approach: IRMA, the iterative refinement meta-assembler. BMC Genomics. 2016;17:708. PubMed

3. Minh BQ, Schmidt HA, Chernomor O, Schrempf D, Woodhams MD, von Haeseler A, et al. IQ-TREE 2: new models and efficient methods for phylogenetic inference in the genomic era. Mol Biol Evol. 2020;37:1530–4. PubMed
